# Draft Genome Sequence of the Extended-Spectrum β-Lactamase-Producing Escherichia coli Isolate INF13/18/A, Recovered from Kelantan, Malaysia

**DOI:** 10.1128/MRA.01497-19

**Published:** 2020-08-13

**Authors:** Wan Ratmaazila Wan Makhtar, Mawaddah Mohd Azlan, Nor Haslizai Hassan, Ismail Aziah, Nurul Hidayah Samsurizal, Nik Yusnoraini Yusof

**Affiliations:** a Reconstructive Sciences Unit, School of Medical Sciences, Universiti Sains Malaysia, Kubang Kerian, Kelantan, Malaysia; b Institute for Research in Molecular Medicine (INFORMM), Health Campus, Universiti Sains Malaysia, Kubang Kerian, Kelantan, Malaysia; c Department of Plant Science, Kulliyyah of Science, International Islamic University Malaysia, Bandar Indera Mahkota, Kuantan, Pahang, Malaysia; University of Maryland School of Medicine

## Abstract

We describe here the draft genome sequence and basic characteristics of Escherichia coli isolate INF13/18/A, which was isolated from Universiti Sains Malaysia (USM) Hospital. This isolate was identified as an extended-spectrum β-lactamase-producing Escherichia coli strain harboring the antimicrobial resistance genes TEM, CTX-M-1, and CTX-M-9.

## ANNOUNCEMENT

Gram-negative Escherichia coli is one of the most versatile and widely studied species in the world (1). The emergence of pathogenic strains can arise through the virulence factors that have evolved by horizontal gene transfer (2). The prevalence of extended-spectrum β-lactamase (ESBL)-producing E. coli worldwide is a serious problem due to the strains resistant to β-lactam antibiotics and other commonly available antimicrobials (3). In this study, we report the whole-genome sequencing technique that was used to sequence an ESBL-producing E. coli strain isolated from Universiti Sains Malaysia (USM) Hospital.

The clinical sample was collected aseptically and incubated into the Bactec 9240 blood culture system (Becton, Dickinson, USA). Gram staining and biochemical testing (4) were conducted. The production of ESBL was examined using Clinical and Laboratory Standards Institute (CLSI) criteria for ESBL screening and disk confirmation tests (5). The isolate was subcultured into nutrient broth (6) and incubated overnight at 37°C and 200 rpm in an incubator shaker. DNA was extracted from the bacterial isolate using the NucleoSpin tissue DNA, RNA, and protein purification kit (Macherey-Nagel) according to the standard protocol. The study protocol was approved by the ethics committee of the Universiti Sains Malaysia (USM/JEPeM/20030152).

We confirmed the 16S rRNA sequences for this strain using two specific primers of E. coli (7, 8). The PCR results indicated that this strain, possessing the TEM, CTX-M-1, and CTX-M-9 types, was carried out with ESBL-specific primers (8). Sequencing libraries were prepared from the genomic DNA using the Nextera XT DNA library preparation kit and sequenced using the Illumina MiSeq sequencing platform (2 × 251-bp paired-end reads).

The total number of raw reads of forward and reverse sequences was 367,947,173 bp. The paired-end sequencing generated 759,821 reads, with read lengths ranging from 35 to 251. Quality control of the reads was performed using BBDuk (BBTools v36) (http://jgi.doe.gov/data-and-tools/bbtools/), with read-trimming and filtered read length threshold values of 30 and 50 bp, respectively. The filtered and trimmed reads (107,495,280 bp) were assembled *de novo* using SPAdes v3.9.0 (9). The 16S rRNA sequence of ESBL E. coli 13/18/A was used to identify identical sequences using the Basic Local Alignment Search Tool (BLAST) (10) against the nonredundant database from the National Center for Biotechnology Information (NCBI). The 16S rRNA was a 100% match with multiple E. coli isolates. The complete genome sequences of E. coli O157 strain AR-0427 (GenBank accession no. CP044148.1) and E. coli strain AR216.2b (GenBank accession no. CP043942), which were the top two hits, were selected to scaffold the draft genome using a graph-based approach with Medusa v1.6 (11). The assembly result was 5,138,415 bp long, with 70 scaffolds generated and an *N*_50_ value of 2,900,087 bp. The GC content was 51.10%. The genome was annotated using the NCBI Prokaryotic Genome Annotation Pipeline (PGAP) v4.10 (12), which predicted 5,002 genes, including 4,712 protein-coding, 9 rRNA, and 63 tRNA genes. Default parameters were used for all software unless otherwise specified. There were a total of 209 predicted pseudogenes, and it may be interesting to explore their association with the pathogenicity of the isolated bacteria.

### Data availability.

The whole-genome project for the ESBL-producing strain Escherichia coli INF/13/18/A has been deposited in GenBank under the accession no. WRPL01000000 (BioProject accession no. PRJNA593306 and BioSample accession no. SAMN13474918). The raw reads are available under the SRA accession no. SRR10587526.
